# Different views on collaboration between older persons, informal caregivers and care professionals

**DOI:** 10.1111/hex.14091

**Published:** 2024-06-24

**Authors:** Teyler van Muijden, Leonoor Gräler, Job van Exel, Hester van de Bovenkamp, Violet Petit‐Steeghs

**Affiliations:** ^1^ Healthcare Governance Erasmus School of Health Policy & Management Rotterdam Netherlands; ^2^ Department of Health Economics Erasmus School of Health Policy & Management Rotterdam Netherlands

**Keywords:** care triad, collaboration, informal care, older persons care, patient engagement, Q‐methodology

## Abstract

**Background:**

Informal care features high on the policy agenda of many countries to deal with workforce shortages. As a consequence, care provision increasingly takes place in the care triad of care recipients, informal caregivers and care professionals. How collaboration between care partners takes shape depends on how the different partners perceive this collaboration. This paper aims to investigate the relative importance of the different aspects of collaboration from the perspectives of care recipients, informal caregivers and care professionals in the context of the care for older persons in The Netherlands.

**Methods:**

Using Q‐methodology, 32 participants ranked 28 statements that reflect different aspects of collaboration in the care triad and explained their ranking during a follow‐up interview. Participants comprised 9 older persons, 10 informal caregivers and 13 care professionals. Data were analysed using by‐person factor analysis to identify common patterns in the rankings of the statements. Emerging patterns were interpreted and described as views on collaboration using aggregated rankings and qualitative data from the interviews.

**Results:**

Five distinct views on collaboration were found: (1) Emphasizing warm collaboration, (2) trusting care professional's expertise, (3) open and compassionate care professionals, (4) responsive decision‐making by autonomous care professionals and (5) prioritizing care recipient's and informal caregiver's interests. Care recipients and/or informal caregivers were associated with views 1, 3 and, 5, whereas care professionals were associated with all five views.

**Conclusions:**

Our study highlights the importance of recognizing the potential diversity of views between and within different partner groups in care triads. Governmental and organizational policy makers, as well as healthcare professionals who aim to increase or support the involvement of informal caregivers, should take this heterogeneity into consideration.

**Patient or Public Contribution:**

An advisory board of older persons (care recipients and informal caregivers) was involved in the recruitment of the participants, the formulation of the statements and the reflection on the findings of the study and potential implications.

## 1 INTRODUCTION

In many Western countries, care and support services for older persons are under pressure due to high costs and workforce shortages.^1^, ^2^, ^3^ Both in governmental and organizational policies, informal care is presented as a promising way to assist in managing these pressures.^1^, ^3^ The Netherlands can be seen as a case in point. Participation of informal caregivers and older persons is part of a Dutch policy transition from a welfare state to a ‘participation society’.^1^, ^4^ A rationale behind this trend is that if informal caregivers and older persons participate more, care recipients can live at home longer, reducing the burden on care professionals.^5^, ^6^ The workload can also be relieved by transferring care tasks from care professionals to informal caregivers when an older person moves to a care organization. Next to reducing workload, informal care provision is thought to enable more person‐centred care since informal caregivers are more aware of older person's preferences.^1^, ^5^, ^7^ In addition, informal caregivers possess greater flexibility to align with these preferences, as they are less constrained by time limitations, organizational structures or formal protocols.

Due to the increased attention to informal care, conventional perceptions of informal caregivers as visitors to a nursing home and older persons as passive care recipients have evolved. This recent shift acknowledges older persons, informal caregivers and care professionals as collaborative partners in ‘care triads’.^8^, ^9^, ^10^ In care triads, care provision is seen as a communal effort. Each partner's perspective on collaboration shapes the dynamics within these care triads.^7^ Understanding these views is crucial for navigating the complexities of care triads. In this paper, we therefore explore the views of clients, informal caregivers and care professionals on collaboration in care triads for older persons living at home and in nursing homes.

### 1.1 Relational complexity in care triads

Although the need for care triads is increasingly recognized, the functioning of care triads can be challenging in practice.^11^, ^12^ Challenges arise from the inherent complexity of the collaborations within care triads, which stems from the heterogeneity of partners who can have different perspectives on the collaboration.^12^ The different and sometimes conflicting perspectives, arising from diverse interests, needs and expectations, can cause friction or dilemmas in collaboration.^4^, ^13^, ^14^, ^15^ Current studies that explore the different perspectives on collaboration within care triads tend to focus on a singular perspective; either on the perspectives of care professionals,^1^, ^4^, ^5^ older persons^16^, ^17^ or informal caregivers.^18^, ^19^ Combined, these studies illustrate that partner groups can perceive collaboration in the care triad differently. Where older persons and informal caregivers focus more on the process of collaborative care, professionals emphasize the care recipient's outcomes in their collaborative efforts.^1^, ^5^ In relation to the process, older persons address the importance of reciprocity^20^ and autonomy^21^ and informal caregivers underscore the importance of trust and open communication.^8^, ^19^ In addition, different views exist on the roles of older persons and informal caregivers in the collaborations of the care triad.^11^ While informal caregivers explicitly address the importance of valuing their role in the care triad,^18^, ^22^ care professionals sometimes view the role of informal caregivers as burdensome.^1^, ^23^ By studying these partners' perspectives separately, limited attention is paid to how different perspectives relate and can sometimes be in conflict. The few studies that do take the perspectives of all three partners into account, tend to focus on particular aspects of collaboration. They, for instance, focus on shared decision making,^24^ care planning,^25^, ^26^ patient‐centred care^5^ or patient involvement.^11^ By studying these different elements of collaboration separately, attention to how these elements can be valued differently is restricted. Yet, not only can the relative importance of these elements differ among partners but the different elements can also be in conflict.^4^, ^13^, ^27^

This study aims to explore different views on the collaboration of those involved in the care triads of older persons in The Netherlands. As a theoretical lens, we used the ‘Senses Framework’ of Nolan et al.^28^, ^29^ We chose this framework because of its relational view on collaboration. Various authors put forward that collaboration in the care for older persons is not solely instrumental in nature,^27^, ^29^, ^30^, ^31^ but very much relational involving emotional and personal elements. We previously discussed that within care triads relationships arise between different partners who can hold different, sometimes even opposing views.^32^ Nolans' framework comprises six senses or conditions for creating and maintaining supportive relationships, namely a sense of security, belonging, continuity, purpose, achievement and significance. The underlying assumption of the framework is that good care can only be achieved when each partner in the care triad experiences positive relationships that promote these senses. Enhancing our understanding of the relative importance of different aspects of collaboration arising from the senses, and how partners may value these differently, can contribute to lessons for improving collaboration in care practice and for policies aimed at increasing or supporting the involvement of informal caregivers in the care of older persons.

## 2 MATERIALS AND METHODS

The current study was part of a larger research project aimed at developing, implementing and evaluating tools to support the collaboration within care triads in the care for older persons in The Netherlands.^33^ The research was conducted at four organizations delivering care services for older persons with somatic and/or psychogeriatric problems.

To explore the views on collaboration in care triads, a Q‐methodology study was conducted. Q‐methodology is a mixed method technique for the systematic study of subjective points of view, such as opinions, values or beliefs about a particular topic.^34^ In previous studies, Q‐methodology has been found to be useful for exploring the opinions of older persons,^35^, ^36^ informal caregivers^37^ and care professionals.^38^, ^39^ In this study, the checklist of Dieteren et al.^40^ for reporting a Q‐methodology study has been followed. This study was conducted in three steps: (1) development of the statements, (2) recruitment of participants and data collection and (3) data analysis and interpretation. These steps are described in more detail below.

### 2.1 Development of statements

To properly measure opinions on a topic, the set of statements should include all aspects that could be potentially relevant to participants' opinions. In other words, the set of statements should cover a broad range of aspects relevant to the topic. To develop a set of statements for opinions about collaboration in the care triad, existing theoretical models on collaboration, relationships and quality of care in health and social care for older persons were studied (e.g., Senses Framework, Values of Integrated Care, The TRANSCIT Model, Quality Framework for Nursing Home Care). We chose the ‘Senses Framework’ by Nolan et al.^28^, ^29^ as a starting point for the development of the statement set because this framework covers all aspects of collaboration put forward by the other models. Based on the Senses Framework, an overview of relevant aspects of collaboration was extracted and categorized for each of the six senses, and an initial long list of statements was formulated. Subsequently, the long list was discussed extensively within the research team and compared with existing empirical data of the research project on the views of care recipients, informal caregivers and care professionals. This empirical data consisted of interviews, focus groups and observations. We decided to add two statements regarding formal rules to the set, namely statement numbers 12 and 13 (#12, that rules can be deviated from and #13, clarity on the rules of the organization). Thereafter, the long list was discussed with the advisory board of the project consisting of quality assurance and policy officers of the four care organizations and representatives of older persons and an informal care association. They assessed the statements based on completeness, comprehensibility and clarity. Subsequently, the statements were refined by the research team in an iterative process into a set of 28 statements (see Table 1). Finally, the interview materials (statement set, sorting grid, instructions) were assessed among care professionals, informal caregivers and older persons in a similar interview setting as intended during the main study. The participants were asked to evaluate the completeness, comprehensibility and clarity of the materials as well as the feasibility of the ranking exercise. Based on this pilot, no additional adjustments to the materials were made.

**Table 1:** Complete list of statements and composite factor scores.

		Views				
Statements		Emphasizing warm collaboration	Trusting care professional's expertise	Open and compassionate care professionals	Responsive decision‐making by autonomous care professionals	Prioritizing care recipient's and informal caregivers' interests
1	Attention to the knowledge of the care recipient and informal caregiver have	+3	+3	+3	+6	+5
2	Attention to the life story of the care recipient	+2*	+4	+6	+5	+6
3	Attention to care that the care recipient has previously received	+2	+3	+4	+3	+3
4	Have understanding for each other	+5	+4	+6	+4	+4
5	Be accessible to each other	+4	+4	+2	+4	+3
6	That care recipient and informal caregiver are satisfied with their own contribution	+4	+1	+2	+5	+7*
7	That the care professional is also there for the informal caregiver	+4	+3*	+5	+4	+6*
8	That contact remains businesslike	+1	+1	+4*	+1	+2
9	That everyone can make their contribution	+3	+2	+5*	+3	+3
10	That everyone can bring their own knowledge and skills to the table	+4	+5	+3	+4	+4
11	That everyone keeps their agreements	+6	+6	+5*	+1*	+6
12	That it is allowed to deviate from the rules	+2	+3	+1	+7*	+2
13	Clarity about the rules of the organization	+4	+5	+4	+2	+3
14	Clarity about responsibilities	+2	+5	+4	+3	+4
15	Able to make own choices	+4	+5	+3	+6	+2
16	Support each other	+5*	+2	+4	+3	+1*
17	Trust each other	+6	+4	+7*	+5	+4
18	Appreciate each other	+6	+4	+5	+7	+3
19	Able to set boundaries	+5	+6	+3	+4	+2*
20	Prioritize the interests of the care recipient	+3	+7	+5	+5	+7
21	Be open and honest with each other	+7*	+6	+7	+6	+5
22	Discuss what the appropriate care is for the care recipient	+5	+6	+4	+4	+6
23	Care professional has control over the care	+3	+2	+2	+2	+1
24	Be considerate of each other	+5	+3	+6	+2*	+4
25	Be willing to learn from each other	+1	+2	+1	+6	+5
26	Trust the know‐how of the care professional	+7	+7	+3	+2	+5*
27	Discuss expectations with each other	+3	+5	+2	+5	+5
28	Feel that you are not on your own	+6	+4	+6	+3	+4

* Factor score is distinguishing for that factor (*p* < .05).

### 2.2 Recruitment of participants and data collection

Care recipients, informal caregivers and care professionals were invited to participate in the study via contact persons at the four care organizations. Targeted recruitment was used to capture a wide range of opinions on what is important for good collaboration within the care triad. This included nurses, caregivers, day‐care coaches, care recipients and informal caregivers (see Appendix S1A). Due to the cognitive load of the study, care recipients were selected whose informal caregivers believed they could perform the task.

Between March and July 2022, a total of 32 interviews (with 9 older persons, 10 informal caregivers and 13 care professionals) were conducted by two researchers (T. v. M., L. G.). Intermediate analyses were conducted to inspect whether the chosen sampling frame led to the expected variety in views and whether participants not loading on any of the factors represented coherent complementary views that needed corroboration through additional data collection. Data collection was stopped after saturation. One interview was terminated because the care recipient was unable to complete the task, the remaining 32 interviews were completed. The interviews took place in the care organizations or at home. Before the interviews, participants signed an informed consent form. Participants were informed about the purpose of the study, the ranking process, the intended use of the data and that they could stop the interview at any time. The protocol for this study has been approved by the Research Ethical Review Committee of Erasmus School of Health Policy & Management (file 21‐028).

**Figure 1:**
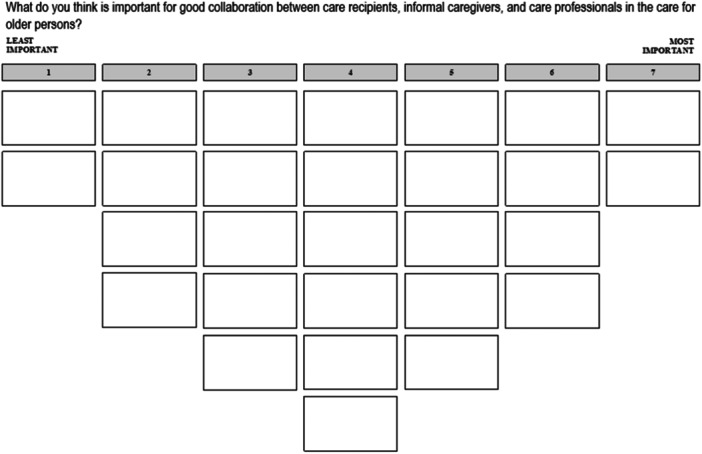
Sorting grid.

At the start of the sorting task, participants were presented with the following research question: ‘What do you think is important for good collaboration between care recipients, informal caregivers and care professionals in the care for older persons?’ To familiarize participants with the set of statements and reduce the cognitive load of the task, participants first read the 28 cards containing the statements and divided them into three piles: important, neutral and unimportant for collaboration. Subsequently, they ranked the statements on the sorting grid (Figure 1) by first reviewing the statements in the important pile and placing them in the boxes on the right side of the chart; then the cards in the unimportant pile in the boxes on the left side of the chart; and finally, the cards in the neutral pile in the remaining open spaces in the centre of the chart. After all cards had been ranked and the participants were satisfied with the ranking, an interview followed. During this interview, questions were asked about the reasoning behind the placement of the two least and two most important statements, other (striking) statement placements and observations made by the interviewers during the ranking (e.g., changing positions or thinking longer about certain statements). Finally, participants were asked to summarize in two or three sentences their opinion about what is important for collaboration between care recipients, informal caregivers and care professionals. The interviews were recorded, and notes and photographs were taken by the interviewers for the final ranking of the statements. The audio recordings were transcribed verbatim for analysis.

### 2.3 Data analysis and interpretation

To identify patterns in participants' rankings of the statements, factor analysis (i.e., centroid factor extraction followed by varimax rotation) was performed using the statistical programme PQMethod version 2.35.^41^ Two researchers (T. v. M., J. E.) evaluated the results of the analysis and, based on statistical criteria (i.e., eigenvalue > 1) and two or more participants statistically significantly associated with each factor and a first interpretation of the factors, decided on the best factor solution for the collected data. For each factor, a composite ranking of the statements was determined, representing the average weighted ranking of statements in that factor. This was computed by multiplying the ranking of each statement by participants loading (uniquely) on a factor by their factor loading and aggregating this across these participants. The statements were then ranked according to their average weighted ranking (from the two highest scoring statements ranked in column 7 to the two lowest ranked in column 0), resulting in a composite sort for each factor (see Table 1). To compute the distinguishing statements, the aggregate scores in each factor were standardized (with mean 0 and SD 1) to correct for differences in scores between factors resulting from different numbers of participants loading on them. Subsequently, the factors were interpreted and described as distinct views on what is important for collaboration in the care triad based on the weighted average ranking of the statements per factor (see Table 1) and the interviews with participants associated with each factor. For the quantitative data, the emphasis was on the characterizing statements (i.e., with a score of +7, +6, +2 and +1 in the factor) and the distinguishing statements (i.e., which had a statistically significantly different score than in the other factors). In addition, a qualitative thematic analysis of the transcripts from participants who were statistically significantly associated with the factors (*n* = 19) was conducted. This systematic coding process aimed to identify recurring themes related to the factors, allowing for a deeper exploration of the content from these interviews and verifying interpretations of the views. Finally, the descriptions of the factors were enriched with quotes from the interviews. The results were validated with the members of the advisory board.

## 3 RESULTS

**Figure 2:**
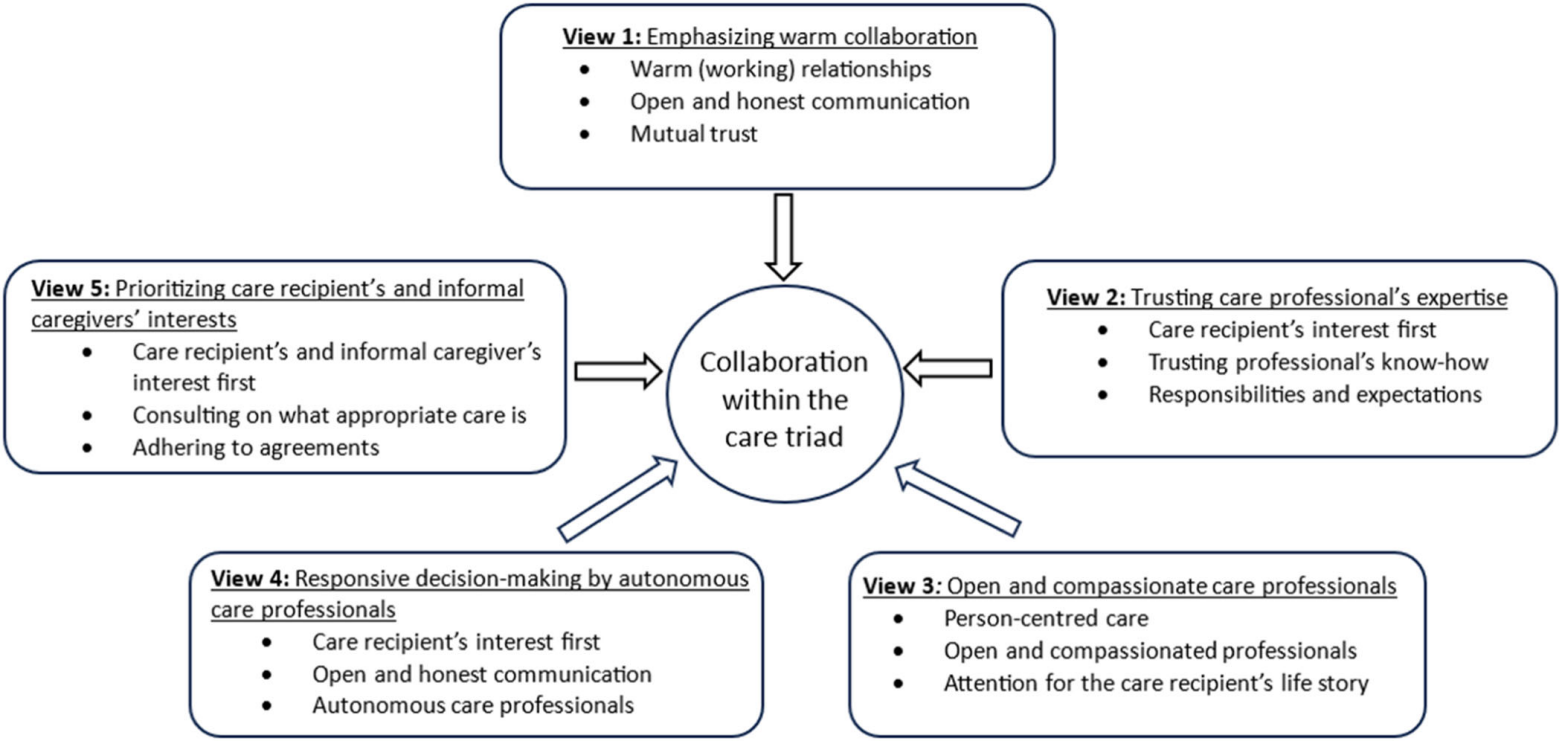
Views on collaboration.

Analysis of the rankings of the statements revealed five views on collaboration between care recipients, informal caregivers and care professionals. Nineteen participants correlated statistically significantly and uniquely with one of the factors (*p* < .05). The other 13 participants either loaded on more than one factor (i.e., confounded, *n* = 6) or did not load on any factor (*n* = 7); their data contributed to the identification of the factors but was not used in their interpretation as views on what is important for collaboration in the care triad. In total, the factors accounted for 50% of the study variance. The analysis revealed a low‐to‐moderate correlation between the factors, ranging between −0.11 and 0.46 (see Appendix S1B). Table 1 shows the composite ranking of the 28 statements for each of the five factors. A score of +7 represents ‘most important’ for collaboration within the respective factor, and a score of +1 ‘least important’. Figure 2 summarizes the interpretation of the five factors.

### 3.1 View 1: ‘emphasizing warm collaboration’

Participants defining this first view on collaboration focus on the process of working together. Collaboration was described by these participants as having a good relationship, where mutual support (#16, +5), the feeling of not standing alone (#28, +6) and being considerate of each other (#24, +5) were considered important. For example, understanding the challenges faced by care professionals, and being flexible when plans deviate from the expected course. A care recipient stated:

Yes, I don't grumble either. Even if they come half an hour late […] I'm not the only one who needs help.

Open and honest communication (#21, +7) was highlighted as essential in this view, preferably in an informal and warm manner (#8, +1). It involved discussing what constitutes the appropriate care for the care recipient (#22, +5) and having mutual understanding (#4, +5). Open and honest communication was thought to cultivate trust among individuals by expressing appreciation for each other (#18, +6). Participants within this view perceived the concept of ‘trust’ as multifaceted. On the one hand, it was about having trust in the care professional's knowledge (#26, +7), especially given the dependence of care recipients and informal caregivers on their expertise, as the following care professional explains:

The people admitted here, give up all their privacy; they literally expose themselves to us. So, then it's of utmost importance that they trust us.

On the other hand, trust was crucial in a more general sense (#17, +6). This encompassed the importance of everyone adhering to rules (#12, +2) and agreements (#11, +6). Notably, in this view, collaboration based on trust did not necessarily prioritize the care recipient's interests (#20, +3) nor the leading role of care professionals (#23, +3).

The emphasis in this view did not focus on discussing expectations with each other (#27, +3) or clarity on the division of responsibilities for care (#14, +2). That participants prioritized the process of collaboration over the substantive was also shown by the fact that learning from each other or delving into each other's knowledge or history was considered less significant (#25, +1; #10, +4; #1, +3; #2, +2), as the main source of trust lies in both formal and caregivers' competence to assess needed care. As aptly formulated by one of the care professionals:

If you trust the caregiver's competence and each other and are open and honest with each other (…), you know what kind of care you can expect and what you think of it, so I think that's the most important thing.

Three care recipients, one informal caregiver and one care professional were uniquely associated (*p* < .05) with this view.

### 3.2 View 2: ‘trusting care professional's expertise’

In contrast to the previous view, this view prioritized the outcomes of collaboration, particularly emphasizing the importance of placing the care recipient's interests at the forefront of the collaborative effort (#20, +7), as a care professional explained:

And then it is very important that you can trust the knowledge of the care professional […] caring together for the care recipient, because that is the other most important goal, to put the care recipient's interests first.

To ensure the care recipient's interests were paramount, deliberation on the right care for the care recipient was essential (#22, +6). Similar to view 1, open and honest communication was considered crucial (#21, +6). However, in this view communication related to discussing expectations (#27, +5), establishing boundaries (#19, +6), clarifying divisions of responsibilities and organizational rules (#14, +5; #13, +5) and adhering to agreed‐upon commitments (#11, +6). If these agreements were not honoured, participants should be able to call each other to account. In this view, a more top‐down form of collaboration was favoured, and trust in the care professional's knowledge was cardinal (#26, +7). Even though the interaction did not have to be businesslike (#8, +1) it related less to relational aspects (#16, +2; #24, +3; #17, +4; #18, +4; #5, +4; #28, +4), as explained by this care professional:

And I also think that there should be an honest talk, like, I expect you to provide the right care for such and such, and I expect this from the informal carer. You can just be honest about that, it doesn't always have to be right, but you can have a good conversation about that.

In this view, the care professional's support for the caregiver was emphasized less (#7, +3), as illustrated by this care professional:

And the informal caregiver, we do try to listen to them and do as much as possible, but that is not always possible […] they do not live here, we don't take care of them.

Since care professionals put the care recipient's interests first based on their care professional knowledge and expertise, little value was placed on the care the care recipient has received earlier (#3, +3). Likewise, little value was placed on mutual learning (#25, +2; #1, +3), supporting each other (#16, +2), that care recipient and informal caregiver were satisfied with their own input (#6, +1), or that everyone contributed to the care (#9, +2).

Three care professionals were uniquely associated (*p* < .05) with this view.

### 3.3 View 3: ‘open and compassionate care professionals’

Like the previous view, the emphasis in this third viewpoint was on the outcome of collaboration. The outcome was seen by the participants holding this view as person‐centred care.

Since the view focuses on person‐centred care, importance is attributed to placing the older person's interests first (#20, +5) and acknowledging the significance of the life story (#2, +6). Following this view, care professionals should be mindful of the importance of caregivers (#7, +5) in the care process.

Notably, control of care did not rest solely with the care professional (#23, +2), nor with the care recipient or caregiver. Hence, less importance was placed on discussing expectations (#27, +2) and setting boundaries (#19, +3). Certain elements, such as making individual choices (#15, +3) and care recipient and informal caregiver satisfaction with their input (#6, +2), were considered less critical due to factors like care recipients' limitations in decision‐making due to psychogeriatric issues and varying levels of involvement from informal caregivers. As stated by a care professional:

Look, I have the care recipients who are from the dementia group, they aren't really aware, they are not really present, so to speak, and the informal caregivers… Yes, I also have a lot of informal caregivers who are not involved.

Compassion from care professionals towards care recipients and informal caregivers was important as a part of the working relationship, even if not always reciprocated. Particularly, aspects like approachability (#5, +2), a willingness to learn from each other (#25, +1) and mutual support (#16, +4) were not the primary focus in this view.

Trust among individuals was of greater significance in this view than in any of the other views (#17, +7), which could be achieved through the care professional's openness and compassion. A care recipient stressed the critical nature of trust: ‘Because you have to trust them, you have to trust these people, if you can't, you shouldn't be here’. To establish and maintain this trust, open and honest communication (#21, +7), mutual understanding (#4, +6) and being considerate of each other (#24, +6) were underscored. These elements were crucial in reassuring that everyone involved is genuinely present for one another (#28, +6) as put forward by the following care recipient.

You have the reassuring idea and feeling that they really say, we are here for you, I think that is important.

Three care recipients and one care professional were uniquely associated (*p* < .05) with this view.

### 3.4 View 4: ‘responsive decision‐making by autonomous care professionals’

Like views 2 and 3, this viewpoint gave precedence to the collaboration's outcome; in this case, placing the care recipient's interests first. To prioritize the care recipient's interest, responsive decision‐making by autonomous care professionals was crucial; namely making independent decisions (#15, +6) and (occasionally) deviating from established rules (#12, +7). This divergence set this view apart from the other four. Drawing on informed judgement, care professionals strategically used rules to manage informal caregivers, either by keeping them at bay or, conversely, by facilitating task accomplishment. The following care professional illustrated the latter care professional:

According to the rules, visitors are not allowed coffee from the department. [‥] And then I think we should be happy with the people who are here, so please give them a cup of coffee or a sandwich or whatever.

Consequently, a reduced emphasis on a clear understanding of organizational rules (#13, +2) was noted. While autonomy for care professionals was pivotal, the control of care did not solely lie with the care professional (#23, +2).

While informal contact among care recipient, informal caregiver, and care professional was preferred (#8, +1), this view, like view 2, placed less emphasis on fostering relationships (#4, +4; #16, +3; #28, +3). This decreased emphasis might be due to the challenge of building relationships in shorter care recipient stays, impacting considerations for others, clarity on the division of responsibilities and strict adherence to agreements (#25, +2; #14, +3; #11, +1). A care professional elaborated on this:

What is difficult lately. […] That people are admitted in an increasingly worse condition since they live at home longer. They don't have a place in the nursing home yet, so they are admitted here first and stay here for 3‐4 months and then move to the nursing home. So, when you just made that contact, built that relationship, they must move on.

Although the focus did not lie on fostering relationships, open and honest communication (#21, +6) was valued, along with the expression of mutual appreciation (#18, +7) and the sharing of expectations (#27, +1). Learning from each other was emphasized (#25, +6), including acknowledging and utilizing the knowledge possessed by both the care recipient and caregiver (#1, +6). Leveraging this knowledge was key to prioritize the care recipient's interests (#20, +5). One care professional shared:

For example, that we say to that informal caregiver: will you teach us? Literally. […]. But vice versa as well. If the informal carer no longer knows how to deal with his mother, who no longer talks, we can respond to that. So yes, and we still learn from the residents every day, by how you deal with the residents every day.

Two care professionals were uniquely associated (*p* < .05) with this view.

### 3.5 View 5: ‘prioritizing care recipient's and caregivers’ interests’

Just like views 2–4, participants defining this viewpoint emphasized the outcome of collaboration. The outcome's primary focus was on ensuring the fulfilment of both the care recipient and the informal caregiver.

Here, paramount importance was placed on the care recipient's interests and life story (#20, +7; #2, +6). Unlike the other views, there was a distinct focus on the caregiver's role in this view. Relocating a loved one to a care facility is seen as profoundly impacting informal caregivers, leading to an emphasis on the care professional's role in supporting them(#7, +6). Furthermore, recognizing the knowledge of the care recipient and informal caregiver is considered important (#1, +6). For example, when care recipients have difficulty communicating, the knowledge held by caregivers is highly valued, as a care professional illustrates:

Regarding people with dementia, you need a lot of knowledge from the informal caregiver. That line must be very short. That needs attention too. It's quite an impact, also for the informal care giver. So yes, I think that is a very important part.

Control over care, unlike other views, was perceived as an interplay involving the care professional, care recipient and caregiver. It must not solely lie in the hands of the care professional (#23, +1), as the following informal caregiver illustrated:

I think that's one, that you have to be involved in that. So that control over care lies not only with the care professional, but with the informal caregiver and, the care recipient as well. […] Because if the control over care lays only with the care professional, well then, many things would not work out.

Within this line of reasoning, satisfaction regarding their contributions to care held a significant position for all three actors (#6, +7). In addition, there was an imperative need for deliberation to determine the appropriate course of care (#22, +6). Establishing clear expectations for collaboration and adhering to agreements were considered indispensable components (#27, +5; #11, +6). The following care professional explained this:

Often it is seen differently, informal caregivers are there for us. No, we are here for them. You can also see that in my ranking of the statements. I think the satisfaction of the care recipient and the informal carer is most important. Of course, you must make certain agreements about that.

Three care professionals, one care recipient and one informal caregiver were uniquely associated (*p* < .05) with this view.

## 4 DISCUSSION

This study aimed to gain a deeper understanding of the relative importance that the partners in the care triad attribute to different aspects of collaboration. This was studied in the context of older persons' care in The Netherlands, an exemplary case in which the need for collaboration in the care triad has been rising. A Q‐methodology study among 32 participants showed five views on collaboration. The first view focused on a warm collaboration based on communication, trust and agreements. The second on older person's interests by trusting the care professional's expertise. The third view emphasized person‐centred care based on openness and compassionate care professionals. View 4 underlined the importance of older person's interests through open communication and responsive decision‐making by autonomous care professionals. And the fifth, and last, view accentuated older person's and informal caregiver's interests through consultation and interplay.

The five views differ in how the distinct aspects of collaboration are valued. First, the views differ in their focus on either the process or the outcome of collaboration. Where view 1 centres on a symbiotic collaborative process, the other four views place more value on the outcome of collaboration. It is noteworthy that views 2–4 prioritize the interests of older persons, whereas view 5 places equal emphasis on both the older person's and informal caregiver's interests. Among the views that centre on the outcome for the care recipient, view 3 prioritizes a more holistic approach by focusing on the care recipient as a person. Furthermore, these outcome‐oriented views hold contrasting views on how these outcomes could be achieved: either by placing trust in the care professional's expertise (views 1 and 2) or by expecting a more active role from care recipients and informal caregivers (views 3–5). A final difference that emerged was the degree to which adherence to rules was valued. While view 4 values the ability to deviate from rules, the other views consider it important that rules are followed.

Previous studies on collaboration in the care triad indicate distinct views between different partner groups. Our study, however, reveals that some of the viewpoints on collaboration are shared between partners in the care triad. An example is the extent to which the outcomes for the care recipient are valued. Where past studies attribute the emphasis on the outcomes for the care recipient as a care professional perspective,^1^, ^5^ our findings demonstrate a common prioritization of this aspect by care professionals, informal caregivers and older persons. In addition, more process‐related aspects of collaboration such as trust, autonomy and open communication, attributed to informal caregivers' and older persons' perspectives in previous studies,^19^, ^21^ were found to be shared across different partner groups in our study. Another example is the legitimization of the role of informal caregivers. In existing studies, acknowledging the role of informal caregivers has been exclusively ascribed to the informal caregivers' perspective.^18^, ^22^ The involvement of informal caregivers has even been addressed as burdensome from a care professional standpoint.^1^, ^23^ The results of this study illustrate a more nuanced understanding in which diverse views exist on the legitimization of informal caregivers' role among care professionals, informal caregivers and older persons.

This study's relational approach might explain why views are not always tied to specific roles. The Senses Framework^28^, ^29^ distinguishes relational elements in the collaborative relationship in the care triad but pays limited attention to organizational structures, such as formal protocols and rules. In designing the statement set, two statements (#12, that rules can be deviated from and #13, clarity on the rules of the organizations) were added to the set based on previous empirical findings from the project. These two statements underline the relation between these relational and organizational structures since it is through rules that the organization enters the relationship and exemplifies that organizational rules sometimes get in the way of collaboration. For follow‐up research, it is recommended to consider additional structural elements for inclusion in the statement set. In addition to the diversity in which aspects are valued, this study also demonstrates that partners can attribute different meanings to these elements. For instance, centring care around the care recipient can be done in different ways; either by focusing on the care recipient as a person, placing trust in the care professional's expertise, or by maintaining continuous interplay. Similarly, the concept of ‘trust’ takes on slightly different meanings across views. View 3 prioritizes relational aspects of trust (openness, compassion) over technical expertise. Conversely, view 1 encompasses both general trust and trust in professional knowledge, while view 2 prioritizes technical expertise over relational aspects.

In line with Nolan et al.,^28^, ^29^ this study showed that all six senses are relevant for collaboration in the care triad, but further emphasizes that partners can value senses to different degrees and can interpret them in different ways. When comparing senses and view interpretations, all five senses appeared in at least one view, with varying prominence. Importantly, no view excluded a sense entirely, nor did any view equally emphasize all. Each view emphasizes three or four senses, and all senses appear in two or three views (Table 2). However, these connections are only indicative, highlighting the most prominent senses within each view. In reality, all senses contribute to each view to varying degrees.

**Table 2:** Main connections between the senses and the views.

	Sense					
Views	Security	Continuity	Belonging	Purpose	Achievement	Significance
Emphasizing warm collaboration						
Trusting professional's expertise						
Open and compassionate professionals						
Responsive decision‐making by autonomous professionals						
Prioritizing care recipient's and informal caregivers’ interests						

Differences in the relative importance attributed to different senses may lead to difficulties in the collaboration process. For example, when one person considers the outcome—the care recipient's interests—to be paramount (view 2) while the other considers it important to pay attention to the collaborative process (view 1). This divergence in view may result in conflicts, such as disagreements over specific care decisions, misunderstandings about the intended approach to collaboration and conflicting expectations regarding the roles and responsibilities within the care triad. The fact that care professionals are usually engaged in multiple triads at the same time, further adds to the complexity of collaborating in care triads. Consequently, care professionals must constantly switch between relationships with older persons and informal caregivers who hold different views on collaboration.^12^ Also, each care triad may comprise different care professionals and informal caregivers, making it even more challenging for partners in the care triad to assume a role that acknowledges and accommodates all views involved. Previous studies revealed that beneath these different needs and experiences often lie diverse values and that these values may come into conflict.^42^, ^43^ As such, the intricate relationships underscore the potential for conflicts, emphasizing the complexity that partners face in navigating collaboration in care triads.

### 4.1 Strengths and limitations

Our study aimed to explore views on collaboration in care triads, not the dynamics within care triads. Investigating views within triads over time could offer deeper insights into these internal dynamics and their evolution. Furthermore, in recruiting participants, we were not able to include older persons with severe dementia or older persons and informal caregivers who are not proficient in the Dutch language. As a result, alternative views on collaboration in the care triad may have been missed. Future studies incorporating relevant subgroups of care recipients and informal caregivers may reveal additional views or add nuance to the five views identified. Another limitation of this study is that it can be seen as a snapshot of the current situation. Views on collaboration can change over time as well as be based on context, for instance, because of the increasing shortage of staff or implementing policies to support collaboration. Notwithstanding these limitations, our study encompassed all three partner groups within the care triad, which helped us to provide a more nuanced understanding of differences and commonalities in how different elements of collaboration are valued, and the potential opportunities and threats these hold for collaboration.

### 4.2 Recommendations for policy and practice

Our study highlights the importance of recognizing the complexity of relationships within care. This complexity receives insufficient attention in policy documents that shape the work of care professionals, encompassing competency profiles and quality frameworks.^43^ A policy stance that is overly rigid in defining collaboration, solely focusing on one viewpoint, may therefore obstruct the alignment between various viewpoints and potentially cause friction in the collaborative effort. Hence, policy frameworks should be designed to foster adaptive efforts in establishing collaborative relationships with various partners in the care triad who can hold different views.^1^ To understand and raise awareness about the variety of views and how these can lead to different expectations, open and continuous dialogue between partners in the care triad is essential.^44^ This dialogue not only serves as a conduit for understanding but also lays the foundation for building trust among stakeholders. Integral to fostering open and continuous dialogue is the cultivation of reflexive and communication skills among care professionals.^45^ Moreover, these skills are considered equally valuable for care recipients.^46^ Furthermore, the provision of tools to support all partners in the care triad in navigating the potentially different views is crucial for collaboration. Open communication and communication skills, fostered through the tools and perspectives developed in our study, can help bridge these gaps, build a mutual understanding of what constitutes good care and enhance collaboration. Achieving alignment between partners necessitates dedicated time and resources to nurture relationships. Increased awareness and a reframing of this collaboration are crucial.^7^ This starts with acknowledging the existence of collaboration in care triads, which was often overlooked. Importantly, this also means that increasing the involvement of informal caregivers in the care for older persons and realizing the advantages expected from their involvement (i.e., saving care professionals' time) will not happen spontaneously but requires acknowledgement and effort from all partners involved at both the care and the policy level.

## 5 CONCLUSION

This study found five views on what is important in the collaboration among partners involved in care triads for older persons. Policy on informal care needs to acknowledge this variety of views and support care professionals, care recipients and informal caregivers to effectively navigate the different views they encounter to improve collaboration. Policy recommendations include acknowledging the diversity of views and advocating for adaptive collaboration through flexible policy frameworks. Recommendations for practice involve fostering reflexivity and open and continuous dialogue to understand and raise awareness of views within the care triad.

## AUTHOR CONTRIBUTIONS

**Teyler van Muijden**: Investigation; writing—original draft; formal analysis; conceptualization; methodology; writing—review and editing. **Leonoor Gräler**: Writing—original draft; writing—review and editing; investigation. **Job van Exel**: Conceptualization; funding acquisition; writing—review and editing; methodology; formal analysis; supervision. **Hester van de Bovenkamp**: Writing—review and editing; writing—original draft; supervision; project administration. **Violet Petit‐Steeghs**: Writing—original draft; writing—review and editing; supervision; project administration.

## CONFLICT OF INTEREST STATEMENT

The authors declare no conflict of interest.

## ETHICS STATEMENT

The protocol for this study has been approved by the Research Ethical Review Committee of Erasmus School of Health Policy and Management (file 21‐028). Participation was voluntary and written consent was collected from each participant before interviews. The data material was saved securely and ensured to be not relatable to specific individuals upon publication.

## Supporting information

Supporting information.

Supporting information.

## Data Availability

Data are available upon reasonable request. All data are in Dutch.
