# Supplementary material for: Different views on collaboration between older persons, informal caregivers and care professionals
Source: Health Expect. 2024 Jun 24;27(3):e14091. doi: 10.1111/hex.14091 (PMC11196834; doi:10.1111/hex.14091)
Supplement: Supplementary file 1 — Supporting information. [file HEX-27-e14091-s001.docx]

Appendix A. Participant characteristics

|  |  | **N** | **%** |
| --- | --- | --- | --- |
| Care recipients |  | 9 |  |
| Gender | Male | 3 | 33 |
|  | Female | 6 | 67 |
| Housing | At home | 4 | 44 |
|  | Nursing home | 5 | 56 |
| Informal caregivers |  | 10 |  |
| Gender | Male | 2 | 20 |
|  | Female | 8 | 80 |
| Care professionals |  | 13 |  |
| Gender | Male | 5 | 38 |
|  | Female | 8 | 62 |
| Role | Nurse | 4 | 31 |
|  | Day-care coach | 3 | 23 |
|  | Care giver | 5 | 38 |
|  | Geriatrician | 1 | 8 |
