# Supplementary material for: Different views on collaboration between older persons, informal caregivers and care professionals
Source: Health Expect. 2024 Jun 24;27(3):e14091. doi: 10.1111/hex.14091 (PMC11196834; doi:10.1111/hex.14091)
Supplement: Supplementary file 2 — Supporting information. [file HEX-27-e14091-s002.docx]

| View | *Emphasizing warm collaboration* | *Trusting care professional’s expertise* | *Open and compassionate care professionals* | *Responsive decision-making by autonomous care professional* | *Prioritizing care recipient’s and informal caregivers’ interests* |
| --- | --- | --- | --- | --- | --- |
| *Emphasizing warm collaboration* | 1 | 0.23 | 0.18 | 0.37 | 0.07 |
| *Trusting care professional’s expertise* |  | 1 | -0.07 | 0.10 | -0.11 |
| *Open and compassionate care professionals* |  |  | 1 | 0.21 | 0.43 |
| *Responsive decision-making by autonomous care professionals* |  |  |  | 1 | 0.46 |
| *Prioritizing care recipient’s and informal caregivers’ interests* |  |  |  |  | 1 |

Appendix B. Correlations between the (composite) factor scores
